# Metabolism and Autoimmune Responses: The microRNA Connection

**DOI:** 10.3389/fimmu.2019.01969

**Published:** 2019-08-28

**Authors:** Alessandra Colamatteo, Teresa Micillo, Sara Bruzzaniti, Clorinda Fusco, Silvia Garavelli, Veronica De Rosa, Mario Galgani, Maria Immacolata Spagnuolo, Francesca Di Rella, Annibale A. Puca, Paola de Candia, Giuseppe Matarese

**Affiliations:** ^1^Treg Cell Lab, Dipartimento di Medicina Molecolare e Biotecnologie Mediche, Università degli Studi di Napoli “Federi II”, Naples, Italy; ^2^Dipartimento di Biologia, Università degli Studi di Napoli “Federico II”, Naples, Italy; ^3^Laboratorio di Immunologia, Istituto di Endocrinologia e Oncologia Sperimentale, Consiglio Nazionale delle Ricerche (IEOS-CNR), Naples, Italy; ^4^Fondazione Santa Lucia, Unità di Neuroimmunologia, Rome, Italy; ^5^Dipartimento di Scienze Mediche Traslazionali, Università degli Studi di Napoli “Federico II”, Naples, Italy; ^6^Dipartimento di Senologia, Oncologia Medica, IRCCS–Fondazione G. Pascale, Naples, Italy; ^7^Department of Cardiovascular Diseases, IRCCS MultiMedica, Milan, Italy; ^8^Department of Medicine and Surgery, University of Salerno, Baronissi, Italy

**Keywords:** T cells, metabolic regulation, immunometabolism, miRNAs, autoimmune diseases

## Abstract

Distinct metabolic pathways are known to regulate growth, differentiation, survival, and activation of immune cells by providing energy and specific biosynthetic precursors. Compelling experimental evidence demonstrates that effector T cell functions are coupled with profound changes in cellular metabolism. Importantly, the effector T cell-dependent “anti-self” response characterizing the autoimmune diseases is accompanied by significant metabolic alterations. MicroRNAs (miRNAs), evolutionary conserved small non-coding RNA molecules that affect gene expression by binding to target messenger RNAs, are now known to regulate multiple functions of effector T cells, including the strength of their activation, thus contributing to immune homeostasis. In this review, we will examine the most recent studies that describe miRNA direct involvement in the metabolic reprogramming that marks effector T cell functions. In particular, we will focus on the work showing a connection between miRNA regulatory function and the molecular network dysregulation that leads to metabolic pathway derangement in autoimmunity. Finally, we will also speculate on the possibility that the interplay between miRNAs and metabolism in T cells may help identify novel miRNA-based therapeutic strategies to treat effector T cell immunometabolic alterations in pathological conditions such as autoimmunity and chronic inflammation.

## Introduction

The immune system encompasses a variety of cellular subsets that are highly dynamic and specialized in several activities essential for host defense and tissue homeostasis (1–4). T cells are a crucial component of the adaptive immune system, with a unique nature that makes them able to respond rapidly to environmental changes (5–7). T cell activation and function are deeply related to specific metabolic programs necessary to regulate T cell signaling and support their growth, differentiation, and effector function. Indeed, the ability of intracellular metabolism to integrate signals and nutrients to produce energy is fundamental to determine a specific T cell fate (8–10). Over the past decade, increasing studies have highlighted the role of microRNAs (miRNAs) in the metabolic control of immune cells. miRNAs are a class of small non-coding RNAs involved in the fine-tune regulation of gene expression. Several mechanisms have been uncovered by which miRNAs control T cell function, including regulation of intracellular metabolic pathways. Under specific conditions, such as inflammation, the biogenesis of miRNAs may be modified. Aberrant expression of miRNAs may influence T cell metabolic reprogramming, leading to pathological phenomena, including chronic inflammatory disorders and autoimmune conditions.

In this review, we discuss recent literature reporting the involvement of miRNAs in the metabolic control of immune responses, with particular emphasis on those impacting on T lymphocyte differentiation and function, in health and autoimmunity.

## Metabolic Regulation of T Cell Response

T lymphocytes play a crucial role in host defense and coordination of immune response (11). Upon antigen recognition via the T cell receptor (TCR) in the presence of co-stimulatory signals, T lymphocytes clonally expand and produce cytokines to eliminate infected or transformed cells (12, 13). In particular, during an acute infection, antigen-specific T cells proliferate and differentiate into effector T (Teff) cells: CD8^+^ cytotoxic T lymphocytes (CTLs) that rapidly mediate the clearance of infected cells and CD4^+^ Teff cells that became functional specialized in distinct T helper (Th) cell subset [Th1, Th2, Th17, follicular Th (Tfh), and regulatory T (Treg) cells] (14, 15). Their dysregulated activation leads to a wide spectrum of autoimmune and inflammatory conditions (16–19).

**Figure 1:**
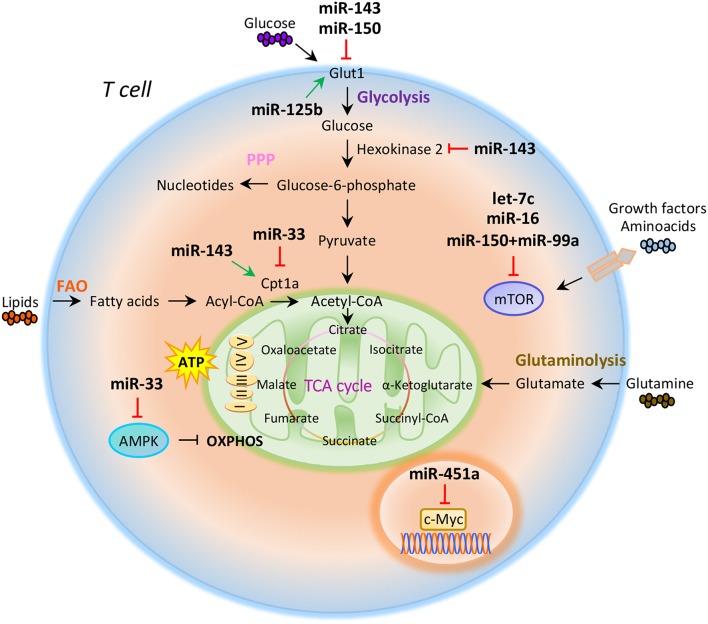
miRNAs orchestrate T cell metabolic reprogramming. Schematic representation of the main metabolic programs controlled by miRNAs in T cells: Glycolysis, pentose phosphate pathway (PPP), fatty acid oxidation (FAO), tricarboxylic acid (TCA) cycle, glutaminolysis, and oxidative phosphorylation (OXPHOS).

Compelling evidence indicate that intracellular metabolic programs adopted by T cells finely regulate immune response (20, 21). T cell activation determines an increased biosynthetic demand, which requires rapid changes to generate metabolic intermediates for T cell growth, proliferation, and function (20–22). Both quiescent naïve and memory T cells are characterized by metabolic pathways that supply energy for survival and migration, including oxidative phosphorylation (OXPHOS), fatty acid oxidation (FAO), and amino acid oxidation [Figure 1; (10, 23)]. Conversely, activation of T cells drives transcriptional changes, causing downregulation of oxidative metabolism and upregulation of biosynthetic pathways, such as aerobic glycolysis that promotes an increase in biochemical intermediates, necessary for nucleotide, amino acid, and fatty acid synthesis (24). This anabolic program increases nutrient uptake at the expense of ATP production (25, 26). Teff cells require high levels of glucose to proliferate and differentiate in distinct T cell subsets (27). The increased glucose metabolism is controlled by glucose transporters, glycolytic enzymes, multi-protein complexes, and transcriptional factors that coordinate glucose utilization to generate pyruvate (28, 29). Under hypoxic conditions, pyruvate can be converted to lactate by the lactate dehydrogenase (LDH) (anaerobic glycolysis); on the other hand, higher oxygen levels promote pyruvate transfer into the mitochondria to supply intermediates of the tricarboxylic (TCA) cycle (30). TCA cycle starts from acetyl-CoA to generate citrate, which is consumed and regenerated throughout multiple biochemical reactions; nicotinamide adenine dinucleotide (NADH), flavin adenine dinucleotide (FADH_2_), and one GTP or ATP molecule are produced in each cycle [Figure 1; (31)]. Subsequently, NADH and FADH_2_ provide electrons to generate ATP via OXPHOS [Figure 1; (31)]. Alternatively, glucose-6-phosphate (G6P), derived from the first enzymatic step of glycolysis, can be directed into the pentose phosphate pathway (PPP) that provides ribose for the synthesis of nucleotides and reducing equivalents, such as nicotinamide adenine dinucleotide phosphate (NADPH), for lipid and cholesterol biogenesis [Figure 1; (32)].

The increased glucose flow into the pentose phosphate pathway is also accompanied by an increase in glutamine metabolism (glutaminolysis), an energy-producing process required for fast biosynthesis of macromolecules necessary for lipid production (33, 34). Lipids play an important role in the regulation and maintenance of membrane properties, bioenergetic demands, and cell signaling (35). Upon TCR activation, T cells must increase their lipid content at each cell division for new plasma membrane generation; moreover, lipids, especially long fatty acids, are necessary to generate energy through the FAO [Figure 1; (36)]. Long-lived memory T cells preferentially use FAO to fuel the TCA cycle and OXPHOS, and maintain ATP production (37). Distinct metabolic and nutrient sensors, including the phosphatidylinositol 3-kinase (PI3K)/AKT/mammalian target of rapamycin (mTOR) pathway, hypoxia-inducible factor 1α (HIF1α), c-Myc, and AMP-activated kinase (AMPK), integrate external stimuli and nutrient availability with intracellular metabolic processes (38–42). mTOR is a serine/threonine kinase with a key role in the regulation of T cell metabolism; it integrates multiple signals in response to nutrients, growth factors, energy, and stress to coordinate immune response upon TCR activation (43, 44). mTOR acts through two main signaling complexes, mTORC1 and mTORC2, which differ for their structure and sensitivity to the inhibitor rapamycin (43–46). PI3K/AKT/mTOR signaling pathway activation induced by TCR engagement, CD28 co-stimulation, or IL-2 receptor leads to an increase of glucose uptake via upregulation of Glucose transporter 1 (Glut1) levels, mediated by mTORC1 complex (45, 47). Instead, mTORC2, which mainly responds to growth signals, controls T cell proliferation and survival by AKT phosphorylation that affects glycogen synthase kinase-3 β (GSK-3β) (48, 49). Moreover, mTORC1 activates glycolytic program in T cells also thanks to the downstream transcription factors HIF1α and its target genes (50). HIF1α is an oxygen sensor rapidly activated under hypoxic conditions, which determines the transcription of target genes including erythropoietin (EPO), vascular endothelial growth factor (VEGF), and glycolytic enzymes (51, 52). HIF1α in turn induces the expression of intermediates required for glycolysis, such as Glut1, in the presence of low oxygen availability (53). HIF1α can also increase glycolysis by inducing the expression of pyruvate dehydrogenase kinase 1 (PDK1), a key metabolic enzyme that favors the conversion of pyruvate to lactate (53). mTORC1 is also responsible for the activation of c-Myc, another transcription factor with pleiotropic effects involved in cell proliferation, growth, and metabolism (38, 54). As a metabolic checkpoint, c-Myc is rapidly activated after TCR engagement and induces the transcription of enzymatic mediators of glycolysis, such as lactate dehydrogenase A (LDHA), pyruvate kinase isoenzyme type M2 (PKM2), hexokinase 2 (HK2), and Glut1 (55). c-Myc also induces the expression of transcription factor activating enhancer binding protein 4 (AP-4) which in turn upregulates the abovementioned glycolytic enzymes (56). Conversely, AMPK drives activated T cells to engage OXPHOS and maintain ATP levels under low glucose concentration (57). AMPK is activated in response to energy deprivation and under stress conditions, such as infections, inflammation, and DNA damage (58). AMPK is also a sensor of energy homeostasis and inhibits energy-consuming metabolism by increasing cellular AMP levels during energy deprivation, favoring ATP production (57, 59). Furthermore, AMPK controls catabolic metabolism through the inhibition of acetyl-CoA carboxylase (ACC), a crucial metabolic enzyme of fatty acid biosynthesis, promoting FAO (59). Under nutrient deprivation, AMPK also inhibits mTORC1 complex activity in T cells (57). Indeed, it has been observed that AMPK loss in naïve T cells upregulates mTOR activity and induces glycolysis (57). In all, these findings suggest that metabolism represents a critical checkpoint for T cell activation and function. Distinct levels of regulation (epigenetic, transcriptional, and translational) control and coordinate this cross-talk to ensure the appropriate energetic status underlying the specific immune cell function.

## miRNA Biogenesis and Biological Function

**Figure 2:**
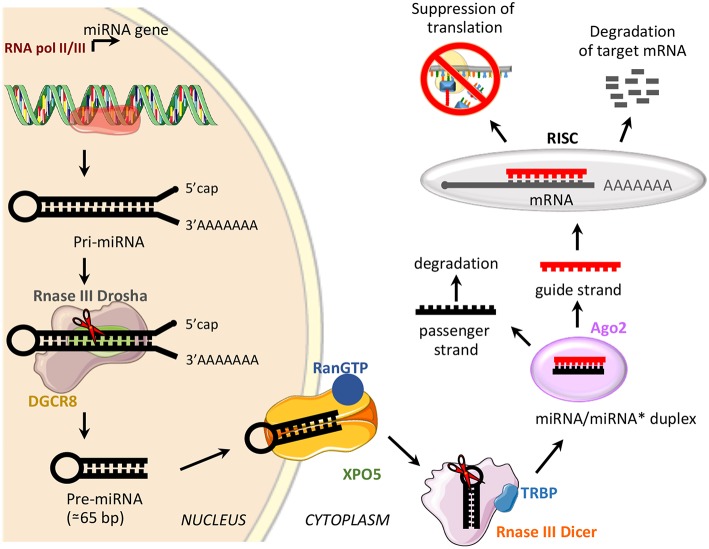
miRNA biogenesis and function. Schematic representation of miRNA biogenesis pathway and biological function. Di George syndrome Critical Region 8 (DGCR8), exportin-5 (XPO5), GTP-binding nuclear protein Ran (RanGTP), trans-activator RNA binding protein (TRBP), Argonaute protein 2 (Ago2), and RNA-induced silencing complex (RISC).

miRNAs are a class of small, non-coding RNAs of 21–25 nucleotides involved in post-transcriptional control of gene expression, through base pairing with complementary sequences in the 3′ untranslated regions (3′UTR) (58). miRNAs are transcribed by RNA polymerase II or III as long variable-length transcripts named primary miRNA transcripts (pri-miRNAs), with a 5′cap and a 3′poly (A) tail [Figure 2; (60)]. Pri-miRNAs are processed by Drosha ribonuclease III and its partner named Di George syndrome Critical Region 8 (DGCR8), to produce a hairpin RNA of about 65 nucleotides known as pre-miRNA molecule [Figure 2; (61)]. After nuclear processing, the pre-miRNAs are exported from the nucleus to the cytoplasm by the GTP-binding nuclear protein Ran (RanGTP)/exportin-5 (XPO5) complex and cleaved into a miRNA duplex of about 21 nucleotides (ds-miRNAs) by a second endoribonucleolytic Dicer and its cofactor TRBP (trans-activator RNA binding protein) [Figure 2; (62)]. ds-miRNAs are loaded into the Argonaute protein (Ago2), which facilitates incorporation of the guide strand into the RNA-induced silencing complex (RISC), while the passenger strand is degraded [Figure 2; (63)]. The guide strand binds target messenger RNA (mRNA) based on sequence complementarity; base pairing match induces degradation of target mRNA, while the imperfect complementarity results in suppression of translation [Figure 2; (64)].

The biological relevance of miRNAs is highlighted by the discovery that a single miRNA could interact with hundreds of target mRNAs, and each gene transcript may have several sites of miRNA recognition (65, 66). The finely tuned control of gene expression requires the production of the appropriate level of specific miRNAs in a well-defined time frame (65, 66). Since miRNAs are involved in post-transcriptional regulation of several cellular processes, dysregulation or dysfunction of their biogenesis leads to a wide range of human diseases, ranging from cancer to autoimmune disorders (67–69). Specific miRNAs have been described to regulate the function and homeostasis of several immune cell populations (70). Compelling evidence has demonstrated that deletion of Drosha or Dicer within T cell compartment affects T cell development, differentiation and function (71, 72). In this context, it has been shown that Dicer deletion at an early stage of T cell differentiation compromises the survival of TCR alphabeta (α/β) chain cells while it is dispensable for CD4 or CD8 T cell lineage commitment (73, 74). Furthermore, Chong et al. highlighted the essential role of Drosha and Dicer in Treg cells, as specific deletion of one or both of them results in impaired expression of the transcription factor forkhead box P3 (FoxP3), the master gene of Treg cell development and function (71). Among all the miRNAs involved in the control of T cell fate, miR-125b has been shown to restrain the expression of genes encoding molecules important for differentiation of naïve into effector and memory T cells, targeting interferon-γ (IFN-γ), interleukin 2 receptor β (IL-2Rβ), interleukin 10 receptor α (IL-10RA), and Blimp-1 (PRDM1) genes (75).

miR-214 and miR-182 act through different mechanisms to control T lymphocyte activation (76). Specifically, up-regulation of miR-124 in T cells, after TCR stimulation, promotes T cell activation through the inhibition of phosphatase and tensin homolog (PTEN), a negative regulator of T cell activation. Mechanistically, activation via CD28 induces upregulation of miR-214, which targets the 3′UTR of *Pten*, causing a reduction in PTEN levels promoting T cell activation (76). Constitutive overexpression of miR-182 by IL-2 supports clonal expansion of Th cells, by reducing the transcription factor Foxo1, a suppressor of resting Th cell proliferation (77).

Compelling experimental evidence reports that several miRNAs can also influence T cell differentiation (78–80). In more detail, miR-155, expressed at high levels in Treg cells, is involved in the modulation of the suppressor of cytokine signaling 1 (SOCS1) and the signal transducers and activators of transcription 5 (STAT5) activity, key molecules for Treg cell differentiation and function (81). Data from animal models revealed that mice lacking miR-146a are more susceptible to chronic inflammation and autoimmune disorders secondarily to the loss of peripheral T cell tolerance. These findings suggest that miR-146 may be considered a key regulator of T cell response (82). In all, miRNA network can be considered as a crucial regulator of cell biology and, although studies focusing on miRNAs are progressively increasing, their impact on T cell function is still only partially explored.

## Role of miRNAs in T Cell Metabolic Reprogramming

The role of miRNAs in the regulation of metabolic reprogramming in cancer cells has been largely investigated, thus opening the study of their potential role in the modulation of T cell metabolism (83–85). Several studies have suggested that miRNAs mainly act on key metabolic enzymes or transporters of energetic nutrients, thus impacting on T cell proliferation and differentiation (86, 87). In this context, Zhang et al. identified miR-143 as a regulator of T cell metabolism that reduces glucose uptake through the inhibition of glucose receptor Glut1. The authors found that, by specifically reducing glycolysis during TCR-dependent activation, miR-143 promotes memory T cell differentiation and metabolic reprogramming (88). In addition, miR-143 stimulates T cell memory differentiation also through the control of two enzymes, hexokinase II and carnitine palmitoyltransferase 1a (Cpt1a), which regulate glucose oxidation and oxidative phosphorylation, respectively [Figure 1; (88)]. miR-150 is an additional miRNA that finely tunes T cell glycolytic reprogramming, secondarily to co-stimulatory signals. Recent findings suggest that CD46 signaling, which is activated downstream of CD28 co-stimulation during T cell activation, leads to a rapid downregulation of miR-150 expression, which targets solute carrier family 2 member 1 (SLC2A1) gene, encoding the glucose transporter Glut1 [Figure 1; (89)]. Through a direct interference with Glut1-dependent glycolysis, miR-150 profoundly impacts on T cell activation in the absence of a proper co-stimulatory signal (89). On the contrary, metabolic reprogramming of activated T cells seems to be associated with the upregulation of miRNAs promoting glucose uptake and downregulation of those stimulating catabolic pathways. In this context, Liu et al. have studied the role of miR-125b, overexpressed in T cell acute lymphoblastic leukemia (T-ALL) (90). The authors revealed that miR-125b regulates glucose uptake in T cell via Glut1 by reducing the expression of TNF-α-induced protein 3 (TNFAIP3), which inhibits the activation of nuclear factor k B (NF-kB) [Figure 1; (90)]. These findings suggest that, through the enhancement of glucose metabolism and oxygen consumption, deregulation of miR-125b contributes to abnormal differentiation of T cell in T cell leukemia (90).

It has been reported that CD28 engagement during T cell activation promotes the expression of Cpt1a, a key enzyme for mitochondrial FAO. This process is finely regulated by miR-33 that, in the absence of CD28 signal, attenuates Cpt1a expression, interfering with the metabolic demand central for future recall of memory T cells [Figure 1; (91)]. Several studies have also reported that miRNAs control T cell metabolic reprogramming by targeting important metabolic checkpoints, such as AMPK, mTOR, and c-Myc (92–94). Ouimet et al. unveiled that miR-33 targets AMPK, inducing an unbalance between aerobic glycolysis and mitochondrial OXPHOS (92). miR-33 affects oxidative phosphorylation and induces macrophages M2 polarization through direct targeting of AMPK [Figure 1; (92)]. The same authors revealed that miR-33 inhibition increased macrophage expression of the retinoic acid (RA)-producing enzyme aldehyde dehydrogenase family 1, subfamily A2 (ALDH1A2), and of retinal dehydrogenase enzyme, with subsequent production of RA, which favors differentiation of FoxP3^+^ CD4^+^ T cells (92).

Several other miRNAs are involved in the control of mTOR pathway; in particular, an increased activation of mTOR, leading to S6 and AKT phosphorylation, has been observed in the absence of co-stimulatory signals in CD4^+^ T cells deficient for RNaseIII enzyme Dicer, a key component of miRNAs biogenesis (93). Two miRNAs are involved in the fine regulation of mTOR and Rictor mRNA expression. Specifically, the downregulation of let-7c miRNA and miR-16 in Dicer-deficient CD4^+^ T cells causes TCR signaling amplification and increased IL-2 production, due to overexpression of mTOR and Rictor mRNAs [Figure 1; (93)]. Subsequently, restoration of mTOR and Rictor expression by genetic manipulation determines reduction of IL-2 production at levels sufficient to avoid anergy in response to TCR engagement (93, 94). Thus, let-7c miRNA and miR-16 control the balance between activation and anergy through post-transcriptional control of mTOR components in T cells [Figure 1; (93, 94)].

mTOR pathway is also regulated by miR-150 expressed at high levels during differentiation of naive CD4^+^ T cells into Treg cells; miR-150 efficiently represses mTOR in cooperation with miR-99a, and this promotes Treg cell differentiation [Figure 1; (87)]. In addition, a recent report showed that miR-451a directly targets c-Myc in Jurkat T cells; indeed, miR-451a negatively correlated with c-Myc expression in CD4^+^ T cells from dilated cardiomyopathy subjects. These results suggest a role of the miR-451/c-Myc pathway in CD4^+^ T cell proliferation and activation [Figure 1; (95)].

These data support the idea that, through the modulation of intracellular metabolic programs, miRNAs are able to influence T cell fate and differentiation (96). Understanding the mechanism by which miRNAs target metabolism in T cells may lead to therapeutic strategies for immune-related diseases.

## T Cell Metabolic Alteration in Autoimmunity: the miRNA Connection

Given the impact of miRNAs in the regulation of T cell metabolic programs, aberrant miRNA expression could interfere with T cell fate with consequent loss of immune homeostasis and autoimmunity (97–100). Autoimmune diseases afflict about 7–9% of the worldwide population; in particular, type 1 diabetes (T1D), systemic lupus erythematosus (SLE), and multiple sclerosis (MS) have been increasing in the last few years (101–104). The role of T cell metabolism in the control of immune function and how its alteration could influence immune response during autoimmunity have been well-characterized (24, 105–111). Naïve T lymphocytes are metabolically inactive, and their differentiation is controlled by metabolic reprogramming, involving glycolysis, FAO, and OXPHOS (10, 112–115). It is well-known that activated T cells require high levels of metabolic compounds to maintain their viability and function (57). In particular, it has been shown that glycolysis sustains cytokine production in Teff cells (57); moreover, FAO and OXPHOS play an important role to increase the inflammatory capacity of memory T cells, and this allows a rapid response upon antigen recall (116, 117). Thus, an aberrant metabolic environment could influence the development of inflammation and autoimmune disorder by fueling the differentiation and activation of pathogenic T cells.

miRNAs were shown to regulate metabolism-related genes in T1D, an autoimmune disease characterized by persistent hyperglycemia secondarily to pancreatic β-cell destruction and insulin deficiency (118, 119). Increased glucose levels in the extracellular microenvironment determine a metabolic reprogramming that fuels autoreactive Teff cell activation and IFN-γ secretion (57, 114, 116). Glucose-activated Teff cells upregulate Glut4 and insulin receptor substrate (IRS)-1 on their surface to sustain glycolytic rate and produce pro-inflammatory cytokines and reactive oxygen species (ROS) that promote the autoimmune response (120). In this context, let-7 family of miRNAs, which control several genes involved in glucose homeostasis, insulin resistance, and cell differentiation, has been reported to control important reguatory mechanisms in T1D subjects [Table 1; (121–123)]. Let-7 miRNAs are one of the largest and highly conserved family of miRNAs expressed in T cells, present in multiple copies in the genome; the number of let-7 miRNAs differs between species; for example, in humans, there are 10 mature let-7 miRNAs (124). It has been shown that let-7 miRNAs target multiple genes related to glucose response and the insulin-PI3K-mTOR pathway, such as insulin-like growth factor 1 receptor (IGF1R), insulin receptor (INSR), and IRS-2 but also regulate genes involved in the effector functions of CTLs (i.e., granzyme A, granzyme B, perforin 1, and eomesodermin) (121, 122, 125). In particular, it has been shown that four members of the let-7 miRNAs family (let-7a, let-7e, let-7f, and let-7g) were higher in PBMCs from T1D subjects (126); however, how these miRNAs are involved in T1D pathogenesis is poorly understood. Together, these results suggest that an altered insulin–PI3K–mTOR pathway in T cells of T1D individuals may determine an aberrant glucose uptake as a consequence of a defective regulation of insulin receptor genes. This impaired metabolism could favor the differentiation and activation of pathological Teff cells in T1D patients. Furthermore, serum miR-378 negatively correlates with insulinoma-associated protein 2 (IA2A) and the Zinc transporter 8 (Znt8) autoantibodies in T1D subjects (127). This miRNA is involved in the control of several metabolic processes, such as glycolysis, mitochondrial oxidation, and fatty acid metabolism, through the interference with the expression of mannose-1-phosphate guanylyltransferase (GDP), dimethylarginine dimethylaminohydrolase 1 (DDAH1), LDHA, and carnitine O-acetyltransferase (CRAT) enzymes [Table 1; (127–129)]. One hypothesis is that serum levels of miR-378 could reflect metabolic alteration that promotes differentiation of pathogenic T cells in T1D subjects (Table 1). In this context, Zhou et al. have investigated by *in silico* analysis that 27 miRNAs out of 530 are located in nine human insulin-dependent diabetes mellitus (IDDM) loci associated with T1D susceptibility (130). Among them, miR-16-2, miR-551b, and miR-877 target specific genes involved in the activation of Teff cells, such as CD28, Fas ligand (FasL), and the autoimmune regulator (AIRE), respectively [Table 1; (130)].

**Table 1:** miRNAs involved in T cell metabolic reprogramming, during autoimmune diseases: type 1 diabetes (T1D), systemic lupus erythematosus (SLE), and multiple sclerosis (MS).

**miRNAs**	**Diseases**	**Targets**	**References**
let-7 family	T1D	IGF1R; INSR; IRS-2	(121–123, 126)
miR-378	T1D	GDP; DDAH1; LDHA; CRAT	(127–129)
miR-16-2	T1D	CD28	(130)
miR-551b	T1D	FasL	(130)
miR-877	T1D	AIRE	(130)
miR-26a	SLE	EZH2	(131–134)
miR-633	SLE	AKT1	(135)
miR-766-3p	SLE	IRS-2; PI3K receptor 1	(136)
NovelmiRNA-25	SLE	AMPD-2	(137)
miR-19b	MS	PTEN	(138)
miR-99b-5p	MS	IGF1R; mTOR; AKT1	(138–142)
miR-21	MS	SMAD7	(143–146)

miR-26a is an additional miRNA associated with T cell dysfunction, glucose metabolism, and autoimmune disease development (131, 132). Its expression in T cells is regulated by glucose availability, and it is able to target the epigenetic regulator enhancer of zeste homolog 2 (EZH2), a histone-lysine-N-methyltransferase, well-known to improve effector T cell function by inhibiting Notch signaling repressors [Table 1; (133, 147)]. In SLE subjects, the levels of miR-26a in CD4^+^ T cells negatively correlate with disease severity; this suggests that the reduced miRNA regulation of EZH2, secondarily to an increased glycolytic activity in CD4^+^ T cells, sustains their activation (134, 148). Several defects in metabolic pathways of T cells from SLE subjects have been described, especially those related to mitochondrial dysfunction (149–151). SLE is a complex multifactorial autoimmune disease where loss of tolerance determines the generation of antinuclear antibodies produced by B lymphocytes and tissue damage by autoreactive Teff cells (150, 152). Mitochondrial membrane hyperpolarization occurs in CD4^+^ T cells from SLE subjects, leading to increase of ROS and depletion of ATP and glutathione, determining an impaired T cell activation and cell death (149, 153, 154). Of note, mounting evidence show a role for mTOR as a sensor of mitochondrial dysfunction in Teff cell differentiation during SLE (155, 156). It has been recently reported that miR-633 that targets the AKT/mTOR pathway is significantly reduced in CD4^+^ T cells from SLE-affected subjects, and its expression negatively correlates with disease activity [Table 1; (135)]. In more detail, miR-633 inhibits the AKT/mTOR signaling and increases the induction of several cytokines, such as IL-4, IL-17, and IFN-γ, thus contributing to disease pathogenesis [Table 1; (135)]. Together with the abovementioned study, others reported an increase of miR-766-3p in CD4^+^ T cells from SLE subjects. Potential target genes of this miRNA are IRS-2 and PI3K receptor 1, both involved in the PI3K/AKT/mTOR pathway [Table 1; (136)]. These data suggest that CD4^+^ T cells of SLE subjects have an impaired PI3K signaling, which could affect their differentiation and function, leading to the development of autoimmunity. Recently, Guo et al. reported the upregulation of several miRNAs associated to metabolic pathways in PBMCs from SLE patients, such as NovelmiRNA-25 and miR-1273h-5p (137). NovelmiRNA-25 targets the enzyme adenosine monophosphate deaminase (AMPD)-2 involved in purine nucleobase or nucleotide metabolism by converting AMP to inosine monophosphate (IMP) [Table 1; (137)]. The overexpression of NovelmiRNA-25 associates with a downregulation of AMPD-2 protein in PBMCs from SLE subjects, contributing to AMP accumulation that improved the activation of pro-inflammatory pathways [Table 1; (137, 157)]. Moreover, increased levels of AMP contribute to activate AMPK, a key enzyme for cellular metabolic reprogramming (42). Of note, NovelmiRNA-25 positively correlates with disease activity, suggesting an important role for this miRNA as a biomarker to predict the activation of pathogenic T cells.

The role of miRNAs in the regulation of autoreactive T cell function has been reported also in MS (158–160). MS is the most common chronic inflammatory demyelinating disease of the central nervous system (CNS), characterized by autoreactive T cells able to target myelin-based antigens, leading to demyelinating lesions and neuronal degeneration (161). During MS, the demyelinating process associates with metabolic reprogramming in neuronal cell bodies sustaining chronic inflammation through the release of pro-inflammatory cytokines (162, 163). These events promote the activation of CD4^+^ T cells, which further increase neuronal damage (162). Mounting evidence highlights the close relationship between T cell metabolic alterations and neurodegeneration in MS (105, 164, 165). Impaired glycolytic engagement has been described in Tconv cells from naïve-to-treatment relapsing remitting (RR)-MS subjects during the generation of induced (i) Treg cells, which contributes to loss of immune tolerance (105). In more detail, the glycolytic enzyme enolase-1 accumulates in the nuclei—secondarily to the reduced engagement in the glycolytic cascade—and constrains the induction of FoxP3 expression during the generation of Treg cells (105). Moreover, other key enzymes involved in glycolysis and mitochondrial respiration are reduced in CD4^+^ T cells from RR-MS subjects, such as aldolase, hexokinase 1, Glut1, dihydrolipoamide S-acetyltransferase (DLAT), and dihydrolipoamide S-succinyltransferase (DLST); interestingly, restoration of these enzymes after IFN-β-1a treatment correlates with disease amelioration (165). Several studies also reported that alterations in the PI3K/AKT/mTOR pathway, which controls T cell activation and metabolism, ameliorate the clinical course of MS (166–169). Also, rapamycin—an immunosuppressant drug that inhibits mTOR by destabilizing the mTOR–Raptor complex—controls disease progression in experimental autoimmune encephalomyelitis (EAE) mice by suppressing Teff cell functions (170, 171). In this context, miR-19b and the miR-99 family are associated with the mTOR pathway, affecting effector T cell activation during MS [Table 1; (138)]. By targeting PTEN, the negative regulator of the PI3K/AKT/mTOR signaling pathway, miR-19b enhances mTOR activity sustaining pathogenic Th17 cell development [Table 1; (138)]. Other reports revealed also that the miR-99 family modulates the PI3K/AKT/mTOR signaling pathways. In particular, the miR-99b-5p, a member of this miRNA family, is significantly higher in splenocytes of EAE mice; *in silico* analysis confirmed that it may target multiple genes, such as IGF1R, mTOR, and AKT1 [Table 1; (139–141)]. Combined miRNA and mRNA expression analysis confirmed these data also in human disease; indeed, miR-99b-5p levels are upregulated in PBMCs from pediatric MS subjects (142). These results suggest an important role for miR-99 family, in particular the miR-99b-5p, in T cell activation during MS through a hyper-activation of the mTOR pathway in pathogenic lymphocytes.

Furthermore, recent reports suggest that fumaric acid ester (FAE)—a Krebs cycle intermediate used for MS therapy—induces hypermethylation of the miR-21 locus in CD4^+^ T cells, and this constrains Th17 cell differentiation and function [Table 1; (143)]. In more detail, FAE treatment reduces Th17 cells, by direct hypermethylation of CpG sites spanning the MIR-21 promoter. Several studies have shown that miR-21 is upregulated in PBMCs from MS subjects and also in CNS-infiltrating T cells of EAE mice (144, 145). As a therapeutic tool in MS subjects, FAE selectively reduces miR-21 transcripts in Th17 cells and indirectly increases its target—the small mothers against decapentaplegic homolog 7 (SMAD7)—an inhibitor of their differentiation [Table 1; (144, 146)].

Taken together, these findings support the existence of a cross-talk between metabolic programs and miRNA network in T cells. Through a strong impact on the intracellular molecular pathways that control T cell differentiation and function, miRNA dysregulation leads to an imbalance between autoreactive T cell activation and regulatory function with consequent loss of immunological tolerance.

## Concluding Remarks

Our understanding of the link between T cell metabolism and miRNA expression has substantially increased in the past decade. The ability of several miRNAs to regulate and reprogram metabolic pathways that drive T cell function and differentiation may represent a hallmark to improve the comprehension of the molecular processes underlying the pathogenesis of autoimmune disorders. However, further studies are required to better understand the connection among miRNAs, T cell metabolism, and loss of immunological tolerance. The precise mechanisms by which miRNAs target the genes encoding for enzymes, multi-protein complex, and transcription factors related to T cell metabolism and how their alteration associates with the development of autoimmune disorders remain to be dissected. Considering the increasing important role of miRNAs in the immune homeostasis, therapeutic approaches could represent an innovative way to control the aberrant metabolism sustaining autoreactive T cell clones.

## Author Contributions

All authors listed have made a substantial, direct and intellectual contribution to the work, and approved it for publication.

### Conflict of Interest Statement

The authors declare that the research was conducted in the absence of any commercial or financial relationships that could be construed as a potential conflict of interest. The reviewer SB and handling editor declared their shared affiliation.
